# Speeding up the Detection of Adsorbate Lateral Interactions in Graph-Theoretical Kinetic Monte Carlo Simulations

**DOI:** 10.1021/acs.jpca.3c05581

**Published:** 2023-11-21

**Authors:** Raz L. Benson, Sai Sharath Yadavalli, Michail Stamatakis

**Affiliations:** Department of Chemical Engineering, University College London, Torrington Place, London WC1E 7JE, U.K.

## Abstract

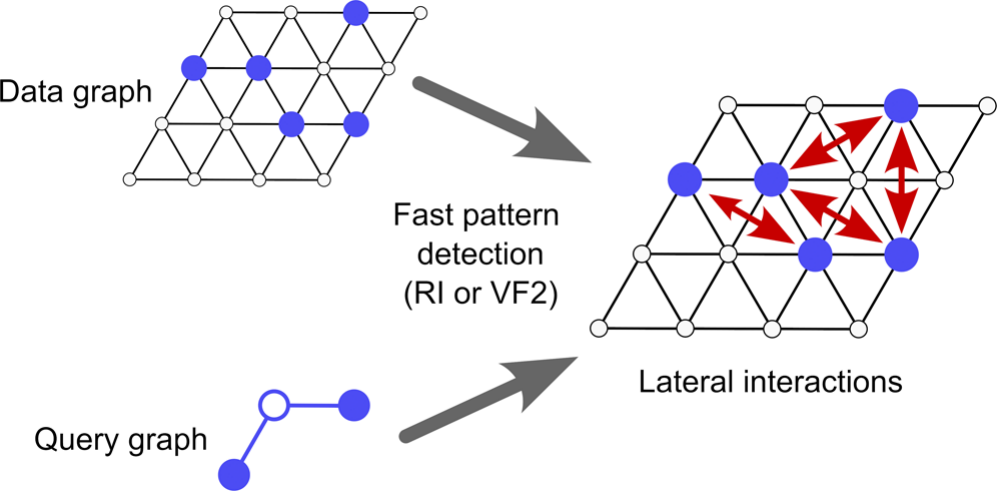

Kinetic Monte Carlo (KMC) has become an indispensable tool in heterogeneous catalyst discovery, but realistic simulations remain computationally demanding on account of the need to capture complex and long-range lateral interactions between adsorbates. The *Zacros* software package (https://zacros.org) adopts a graph-theoretical cluster expansion (CE) framework that allows such interactions to be computed with a high degree of generality and fidelity. This involves solving a series of subgraph isomorphism problems in order to identify relevant interaction patterns in the lattice. In an effort to reduce the computational burden, we have adapted two well-known subgraph isomorphism algorithms, namely, VF2 and RI, for use in KMC simulations and implemented them in *Zacros*. To benchmark their performance, we simulate a previously established model of catalytic NO oxidation, treating the O* lateral interactions with a series of progressively larger CEs. For CEs with long-range interactions, VF2 and RI are found to provide impressive speedups relative to simpler algorithms. RI performs best, giving speedups reaching more than 150× when combined with OpenMP parallelization. We also simulate a recently developed methane cracking model, showing that RI offers significant improvements in performance at high surface coverages.

## Introduction

Kinetic modeling techniques play a pivotal role in the mission to engineer new, more sustainable materials for heterogeneous catalysis.^1−6^ They complement experimental studies by facilitating a detailed understanding of surface reaction mechanisms and accurate predictions of the catalyst activity and selectivity. The “gold standard” of modern kinetic modeling is kinetic Monte Carlo (KMC), a stochastic simulation method that treats elementary chemical processes as discrete events.^7−16^

Formally, an ensemble of KMC trajectories constitutes a numerical solution to the Markovian master equation for the system, which expresses its time evolution in terms of the rates of transitions between a discrete set of memoryless “states”.^8^ In the “on-lattice” variations of KMC used to study catalytic surfaces, each state typically corresponds to one configuration of adsorbates on the two-dimensional lattice.^9^ The memorylessness arises from the assumption that enough time passes between successive events for the current and past configurations to decorrelate. The transitions are the elementary processes, which include adsorptions, desorptions, diffusional hops, and single-step chemical reactions, with rate constants typically estimated using transition state theory (TST).^10^ It is important to recognize that the microscopic TST parameters, which may be determined using density functional theory (DFT) calculations, serve as input to the KMC simulation.

**Figure 1:**
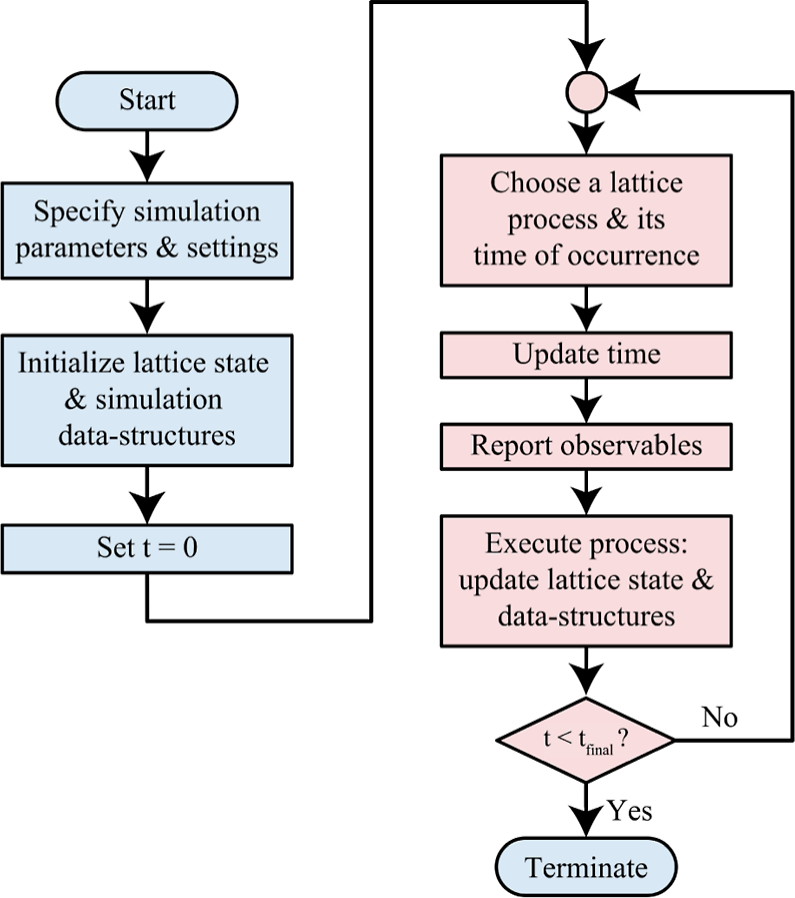
General structure of a KMC algorithm presented as a flowchart. Reproduced with permission from ref (15). Copyright 2022, Authors.

A number of different exact algorithms for KMC are known, some of them under multiple names, but they give statistically equivalent results.^17^Figure 1, taken from ref (15), depicts the general structure common to all KMC algorithms. We will focus in particular on the first-reaction method (FRM), developed originally by Gillespie for well-mixed systems^8^ and then formulated for on-lattice chemistry by Jansen and co-workers.^17−19^ In the FRM, one identifies every possible lattice process that could occur given the current state of the system and places it in a priority queue based on a provisional occurrence time. This occurrence time is sampled from an exponential distribution with the rate parameter being equal to the appropriate rate constant, which may depend on the local configuration. The process of highest priority (earliest occurrence time) is executed, and the state is updated accordingly. This change of state will affect which processes can take place in future and may change their rate constants, so the occurrence times and process queue are also updated as necessary.^8,17−19^

One of the key practical advantages of on-lattice KMC over less expensive microkinetic modeling^20^ is its ability to capture the effects of spatially correlated adlayers in a formally exact way. Spatial correlations may arise even in ideal or near-ideal adlayers, for instance, if the diffusion of one or more adsorbates is slow relative to the reaction.^21,22^ More commonly, however, correlations are attributed to lateral interactions between adsorbates.^23−30^ In KMC, one is at liberty to define the microscopic rate parameters as functions of the adlayer structure, a popular approach to which is to use Brønsted–Evans–Polanyi (BEP) relations that correlate the activation and reaction energies of an elementary event.^31−33^ Thus, lateral interactions may be incorporated explicitly, and doing so has been shown to have a strong effect on predictions of catalytic activity.^25,33−36^

Carrying out high-fidelity (i.e., ab initio) calculations of the microscopic rate parameters (e.g., activation energies) for every relevant configuration of adsorbates on the lattice would be prohibitively time-consuming. One can, however, fit mathematical models of lateral interactions to well-selected ab initio data. A popular approach is to represent the total energy of the lattice and its adsorbates using a so-called cluster expansion (CE), in which each term associates a particular geometric pattern or “cluster” of adsorbates with an effective interaction energy. The problem of computing the adlayer energy is thus reduced to the identification of these patterns on the surface.^33,37−39^ In principle, the CE can represent any adlayer energy exactly, but, in practice, it must be truncated to a manageable number of terms.^40^

Subject to the memorylessness (Markov) property described above, the KMC method is applicable to surface chemistries of almost arbitrary complexity, involving not just lateral interactions but also multiple binding site types and multidentate adsorbate species. In practice, achieving an efficient implementation that can boast such a high degree of generality presents a significant technical challenge. To this end, Stamatakis and Vlachos^10^ formulated a graph-theoretical KMC framework in which the lattice and elementary steps are represented as connected graphs. The elementary steps are then mapped to feasible lattice processes by solving subgraph isomorphism problems, where the elementary step and lattice graphs adopt the roles of the “query” and “data” graph, respectively.^41^ Nielsen et al.^33^ extended the formalism to incorporate the CE framework for adsorbate lateral interactions. Each cluster or “figure” appearing in the CE Hamiltonian has a graph representation and is mapped to lattice sites involved in lateral interactions by solving a subgraph isomorphism problem, analogous to the means of identifying possible lattice processes.^33^ GT-KMC with the FRM is implemented in our Fortran 2003 software package, *Zacros*.^16,33,41−45^

*Zacros* has been used successfully to simulate a range of catalytic systems.^46−60^ Still, while accounting for lateral interactions is possible, it is often extremely time-consuming, especially when the interactions are long-range. Long-range interactions are characterized by the contribution of large clusters to the CE; the cost of solving a subgraph isomorphism problem increases sharply with the number of vertices in the “query graph”, which in this case represents the cluster. Several approaches may be adopted to reduce the computational burden without further approximation, arguably the most straightforward of which is shared-memory parallel processing. Nielsen et al. introduced parallelization in *Zacros* with OpenMP, which was shown to offer considerable speedups depending on the CE employed.^33^ Applied to a model of catalytic NO oxidation on Pt(111), with a particularly challenging CE encompassing up to eighth nearest-neighbor pairwise interactions between adsorbed oxygen atoms (12 figures in total),^32,61^ the parallelization was found to be almost perfectly efficient. The benefit was less pronounced when only shorter-range lateral interactions were considered.^33^ In any case, the speedup will always be limited by the number of threads available as well as the number of clusters that need to be detected. More recently, Ravipati et al. implemented a caching scheme to reduce the number of repetitive pattern detections that need to take place. This works by caching the number of lattice instances of each interaction pattern involving the products of each lattice process, then updating this number efficiently (by detecting only the necessary patterns) when a reaction happens in that lattice process’s neighborhood. Utilizing this scheme in combination with parallel processing was shown to accelerate KMC execution by a factor of up to 20×, using the same 12-figure CE NO oxidation model mentioned above.^42^

A conceptually different approach to evaluating the CE Hamiltonian was proposed by Hess,^39^ in which a look-up table is utilized to specify the interaction energy of a given set of lattice sites as a function of their occupancies. To accelerate the calculations, Hess also developed the supercluster contraction scheme, whereby the number of terms in the CE Hamiltonian is effectively reduced by grouping them together. This requires only inexpensive postprocessing of the original CE and does not introduce any further approximations.^39^ The scheme was introduced in tandem with two other algorithmic improvements, namely, the use of subtraction schemes for updating kinetic parameters and the supersite approach to optimize the selection of the next lattice process. It is important to note, however, that the latter two approaches are applicable specifically to “direct method”-KMC,^8^ also known as the variable step-size method,^19^ whereas *Zacros* implements the first-reaction method as described above. Nonetheless, the supercluster approach shows promise as a standalone acceleration scheme, and further work will be required to determine whether it can be adapted and automated for compatibility with the graph-theoretical CE formalism of Nielsen et al.^33^ Other promising recent advancements include the RMC-MKM approach of Kumar and Chatterjee,^62^ which employs reverse Monte Carlo (RMC) to enhance microkinetic modeling (MKM) with a short-range order parameter that can capture spatial correlations between adsorbates.

Within the context of GT-KMC, another avenue worth exploring is the optimization of the pattern detection algorithms themselves. In *Zacros* 3.01, which is the most recent release, a simple depth-first search (DFS) strategy is used to solve the relevant subgraph isomorphism problems, incorporating rudimentary techniques to reduce the search space based on ideas introduced by Ullmann.^63^ However, subgraph isomorphism is of central importance in a diverse range of fields, including, for instance, cheminformatics,^64^ electronic engineering,^65^ and cybersecurity.^66^ This has driven the development of a large number of alternative algorithms. Notable examples from recent decades include the VF algorithm^67^ and its variants (VF2,^68^ VF2++,^69^ VF3,^70^ and VF3-Light^71^), the RI algorithm,^72^ McGregor’s algorithm,^73^ and focus search.^74^

Which algorithm performs best depends on the properties of the graphs under consideration, such as their sizes, their densities, and whether they are planar. Preliminary investigations using a hexagonal lattice have identified the VF2 and RI algorithms as good candidates for detecting lateral interactions in GT-KMC.^75^ Routines based on these algorithms have now been implemented in *Zacros* as part of this work, alongside the default “refined DFS” (rDFS) solver. For consistency with refs (33) and (42), we assess the performance using the same series of NO oxidation models studied therein. Quite remarkably, for the 12-figure CE, we obtain acceleration factors exceeding 125× by using VF2 or RI in tandem with shared-memory parallel processing. In stark contrast, neither VF2 nor RI offer any benefit (and in fact slow the KMC execution down) when only first nearest-neighbor interactions are considered. We also simulate a more complex system, namely, a model of methane cracking on Ni(111) developed by Yadavalli et al., which involves a 62-figure cluster expansion.^76^ It is shown that RI offers significant speedups when the surface coverage is high such that there are many lateral interaction patterns to detect.

The rest of the paper is organized as follows. Methods contains an overview of the GT-KMC and CE methodology, followed by a discussion of the subgraph isomorphism algorithms under consideration and their implementation in *Zacros*. Results & Discussion details our performance benchmarks, and finally, Conclusions summarizes our findings and their significance.

## Methods

### Theory

Here, we provide an overview of the graph-theoretical KMC (GT-KMC) framework and cluster expansion (CE) formalism for treating adsorbate lateral interactions. For a more detailed discussion, the reader is referred to refs (33), (41), (42).

#### Lattice and Its Coverage

Consider a lattice comprising  possible adsorption sites spanning  site types. The foundation of GT-KMC is a connected, undirected graph, , which represents this lattice. Each vertex  represents a unique site, while each edge  represents a neighboring relation between two sites. The physical nature of site *i* is specified by a three-element vector (formally, a tuple) , where . The elements of **s**_*i*_ denote the site type and the x- and y-coordinates, respectively. The lattice sites can be described collectively by the  array .

To be able to describe the state (coverage) of the system, suppose that there are  different possible surface species (adsorbates). One of these is a vacancy (representing the absence of any adsorbate), and this is given an index of 0. The denticity of species , where , is given by *d*_*n*_ ∈ {1, ..., *d*_max_}, where *d*_max_ is the maximum denticity of all the species (note that *d*_0_ = 1). The state of site *i* is thus fully described by a three-element vector, , where  and  is the total number of distinct entities (adsorbates and vacancies) occupying the lattice sites. The elements of σ_*i*_ specify the entity label, the species, and the subunit thereof adsorbed on site *i*, respectively. The state of the entire surface is accordingly described by the  array . One can easily show that . Note that any observable property of the system must be invariant under permutations of the entity labels, which are entirely arbitrary.

#### Elementary Steps

While **s** does not change, **σ** evolves as the simulation progresses. This occurs via a chemical reaction mechanism consisting of several possible elementary steps, each of which can be represented by a graph. The graph representing elementary step α is denoted by , where the vertices Ξ_α_ = {1,···,*N*_Ξα_} correspond to the sites involved and the edges Ψ_α_ ⊆ (Ξ_α_ × Ξ_α_) describe their connectivity on the lattice. It is important to highlight that the vertices of  do not correspond to any particular set of lattice sites but rather comprise a generic site pattern, instances of which may occur in several locations on the lattice. Unlike for lattice sites, no positional data needs to be specified for the sites of an elementary step, so the nature of site *i* of step α can be fully captured by the scalar variable . The case of ξ_α,*i*_ = 0 arises when the site concerned can be of any type. If necessary, geometric constraints can be defined by specifying the angles between sets of three vertices
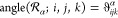
1where *i*, *j*, *k* ∈ Ξ_α_, and 0 ≤ ϑ_*ijk*_^α^ < 2π. The initial and final coverage patterns of step α are fixed properties of , represented by arrays  with *i* ∈ Ξ_α_.

To be able to model the kinetics of the system, one needs to know the propensity of each elementary step. We assume that the kinetic rate constant for step α can be fitted to the Arrhenius equation
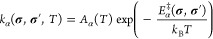
2where *A*_α_(*T*) is the pre-exponential factor, *E*_α_^‡^(**σ**,**σ**^′^) is the activation energy, *k*_B_ is the Boltzmann constant, *T* is temperature, and **σ** and **σ**′ describe the lattice state before and after the reaction, respectively. In practice, *A*_α_ is usually parameterized using TST.^2,77,78^ The activation energy is fitted to a BEP relationship^31^
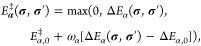
3where Δ*E*_α_(**σ**, **σ**′) is the reaction energy and ω_α_ is the proximity factor. The quantities Δ*E*_α,0_ and *E*_α,0_^‡^ represent the reaction and activation energies in the absence of any spectators (adsorbates not directly involved in the elementary step). The reverse of step α is denoted by α̅ and its activation is given by
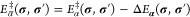
4which stems from the principle of microscopic reversibility.

#### Adsorbate Lateral Interactions

In principle, one could calculate Δ*E*_α_(**σ**, **σ**′) on the fly using ab initio methods, but this would be prohibitively time-consuming. Instead, ab initio (or other) data collected before the simulation can be used to parameterize a Hamiltonian for the adsorbate layer, , which captures the physical and chemical interactions of the adsorbates with the lattice and with each other. Thus,
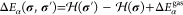
5where Δ*E*_α_^gas^ is the change in energy of all gas species involved in step α.

If there is only one monodentate adsorbed species and one site type, the state vector can be expressed in the simplified form , where σ_*i*_ is equal to 1 if site *i* is occupied or 0 if it is unoccupied. Then a suitable functional form for the Hamiltonian is the lattice-gas expansion^79−81^
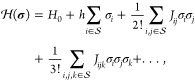
6where *H*_0_, *h*, *J*_*ij*_, *J*_*ijk*_, ... are parameters to be fitted. Specifically, *H*_0_ may be interpreted as the energy of the empty lattice, *h* as an adsorption energy, and *J*_*ij*_ and *J*_*ijk*_ as two- and three-body effective interaction energies. In practice, the lattice-gas expansion is truncated to a finite number of terms. To recast eq 6 in a graph-theoretical form, we recognize that each product σ_*i*_σ_*j*_... evaluating to 1 represents a particular pattern of adsorbates interacting on the lattice, while the coefficient *J*_*ij*..._ gives the associated effective interaction energy. Given the two-dimensional translational symmetry of the lattice, the value of *J*_*ij*..._ must depend only on the geometry of the pattern and not on its absolute position. We can therefore divide the summation into contributions from *F* distinct pattern geometries or “clusters”, also referred to as figures. Each such cluster can be represented by a connected graph , with γ ∈ {1, ..., *F*}. The associated coverage pattern is described by the scalars χ_γ,*i*_ ∈ Φ_γ_ which take values of 0 or 1, with *i* ∈ Φ_γ_. The Hamiltonian can then be expressed as
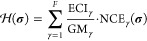
7where ECI_γ_ is the effective cluster interaction for figure γ, GM_γ_ is the graph multiplicity (essentially a symmetry number), and NCE_γ_ is the number of instances of figure γ on the lattice (each of which must have the correct coverage pattern specified by **χ**_γ_). Note that each ECI equates, in general, to a linear combination of the parameters *H*_0_, *h*_*i*_, *J*_*ij*_, *J*_*ijk*_, ... of eq 6.

The advantage of eq 7 is that it immediately generalizes to more complex surfaces, involving  site types and  adsorbate species of arbitrary denticity. The site type of vertex *i* of cluster γ is specified by . A three-element vector **χ**_γ,*i*_ ∈  describes the associated coverage pattern, where & = (&, &, &) denotes a state that is unspecified. This is often the case for intermediate sites in long-range interaction patterns, which can be vacant or occupied by any species; the corresponding graph vertices are dubbed “nonspecific”.^42^ Geometric constraints can be defined where necessary by specifying the angles between sets of three vertices
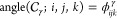
8where *i*, *j*, *k* ∈ Φ_γ_ and 0 ≤ ϕ_*ijk*_^γ^ < 2π.

**Figure 2:**
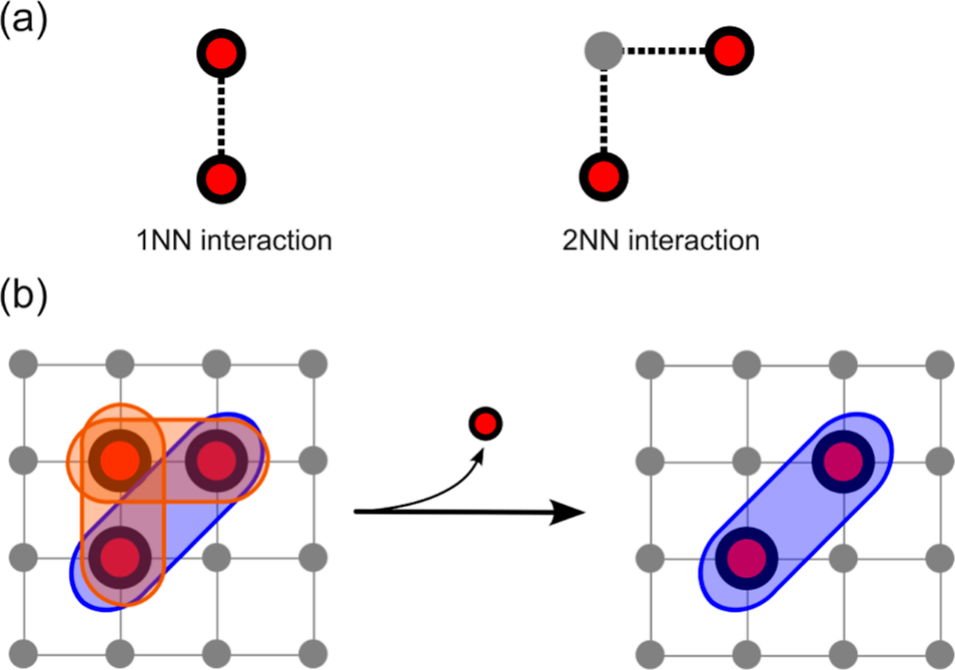
(a) Graphical representation of the 1NN and 2NN lateral interaction patterns contributing to a simplified cluster expansion Hamiltonian for CO on a square lattice. (b) Schematic representation of a CO desorption event with the 1NN and 2NN interactions highlighted in orange and blue, respectively. The desorption event eliminates both 1NN interactions, while the 2NN interaction (in which the central site is nonspecific) remains intact.

By way of illustration, let us consider the energy changes that might occur when a CO molecule desorbs from a square lattice. In our simplified model, the cluster expansion contains just two terms, depicted graphically in Figure 2a. The first involves a 2-site cluster and corresponds to a first nearest-neighbor (1NN) lateral interaction. Both sites are occupied by monodentate CO*; hence, the coverage pattern can be described as
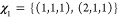
9The second term involves a right-angled, 3-site cluster and corresponds to a second nearest-neighbor (2NN) interaction. While the terminal sites are each occupied by CO*, the state of the central site is unspecified. Hence,
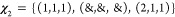
10and the geometric constraint is given by
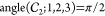
11

Figure 2b depicts the initial and final lattice states for a particular CO desorption event involving two spectators. The relevant 1NN and 2NN interaction patterns are colored orange and blue, respectively. As illustrated, the desorption event eliminates two 1NN interactions; the GT-KMC algorithm must correspondingly remove these from the list of valid pattern instances (see ref (42)). On the other hand, the 2NN interaction remains intact because the intermediate site thereof is nonspecific. In fact, two such “L”-shaped patterns are present on the lattice. However, they correspond physically to the same effective interaction, and only one will be included in the list of valid pattern instances (for nonspecific sites, our algorithms seek only one valid mapping to avoid unnecessary proliferation of interaction pattern instances).^42^

#### Subgraph Isomorphism Problems

A key component of the GT-KMC simulation is compiling the list of potential lattice processes, given the state of the adsorbate layer at a particular time. To achieve this, one needs to identify all chemically sound mappings from the sites of each elementary step to the sites of the lattice. In the language of graph theory, this means finding all unique subgraphs of the lattice graph , that are isomorphic to each elementary step graph .^41^ In this “subgraph isomorphism problem”,  takes the role of the “data graph”, while  takes the role of the “query graph”.^82^

For systems with adsorbate lateral interactions, another key component of the simulation is calculating the lattice state-dependent activation energies using eqs 3 and 4. To do so requires evaluation of the cluster expansion (CE) of eq 7. For each cluster γ, one must count the number of NCE_γ_ of corresponding lateral interactions on the lattice. Similar to detecting lattice processes, this amounts to solving a subgraph isomorphism problem, except that the query graph is now  instead of .^33^ Specifically, we search for each injective function  that meets the following criteria (in which we drop the index γ for brevity):

Crit. 1:

For every edge connecting two sites *i* and *j* of the cluster graph, there is an edge connecting the corresponding sites  in the lattice graph
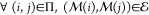
12

Crit. 2:

(a) The type of each site *i* in the cluster graph is compatible with that of its corresponding site  in the lattice graph

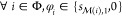
13

(b)The coverage pattern of the cluster graph is compatible with that of its corresponding lattice subgraph

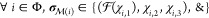
14where
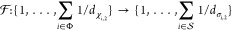
15maps the entities involved in the interaction to those present on the lattice.

(c)Whenever specified, the angles between three vertices of the cluster graph are the same as the angles between the corresponding lattice graph vertices

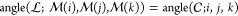
16Taken together, Crit. 2(a–c) can be summarized by the requirement that the vertices of  are *compatible* with those of the corresponding lattice subgraph. It is possible to introduce additional criteria under this heading, such as specifying the absolute orientation of the subgraph with respect to a lattice vector.

Note that the lattice process detection problem is defined by analogous criteria, but we focus on lateral interactions as these are more relevant to accelerating KMC simulations (see below). Note also that in practice, one does not need to identify all possible cluster mappings “from scratch” for every new lattice state. After each event occurs, it is only necessary to calculate activation energies for any newly feasible lattice processes, i.e., those in which the product species participate, as well as any processes already queued that are in the neighborhood of that which just took place. Full algorithmic details of the kinetic constant update steps are described in refs (33) and (42). The key point is that only patterns that involve the relevant product species and any neighboring adsorbates need to be detected to calculate a particular activation energy.

### Algorithms

#### Basic Depth-First Search Algorithms

The subgraph isomorphism problem is well-studied.^63,67−75,82−84^ In the general case, it is NP-complete, with the time taken to solve it scaling exponentially with the size of the query graph.^85^ For specific cases, however, efficient solutions can be obtained via carefully constructed rules and procedures for reducing the search space.

**Figure 3:**
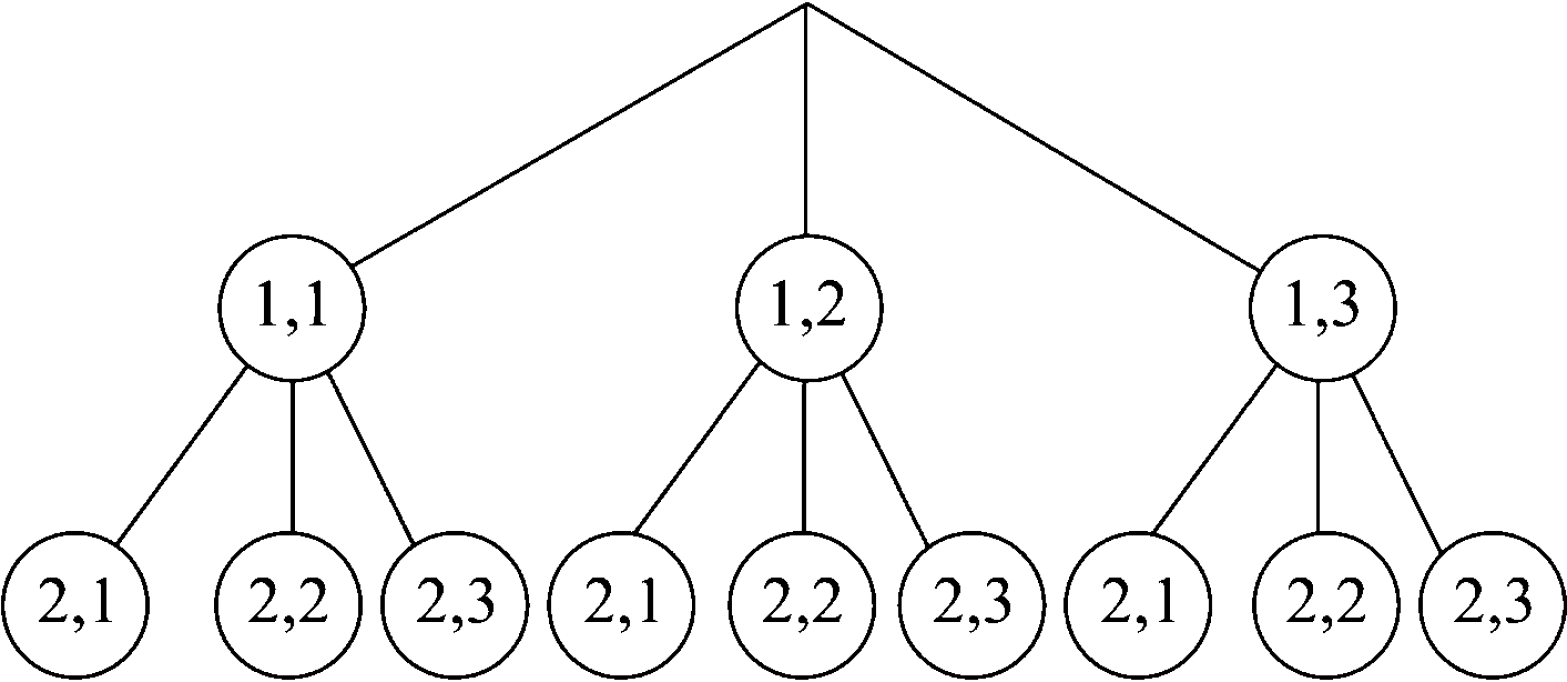
Illustration of the brute-force search tree for subgraph isomorphisms where the query graph contains 2 vertices and the data graph contains 3 vertices. Each node *i*, *j* corresponds to a trial mapping of query vertex *i* to data vertex *j*.

Consider a brute-force matching approach in which one loops over the vertices of the query graph (query vertices) and pairs them, in turn, with each of the vertices of the data graph (data vertices). When every query vertex is paired, this constitutes a trial mapping , where we have adopted the notation of energetic clusters but dropped the index γ for brevity. If  meets both (sets of) criteria detailed above, namely, every query edge has a corresponding data edge and any vertex/edge compatibility rules are obeyed, then it describes a subgraph isomorphism ; otherwise, it is discarded. This procedure is guaranteed to find all subgraph isomorphisms and can be visualized as the traversal of the search tree shown in Figure 3. Each node of the tree labeled *i*, *j* indicates the pairing of query vertex *i* ∈ Φ with data vertex , where, in this case, *N*_Φ_ = 2 and . In general, the tree will contain  branches; thus, the time taken to complete the pattern matching grows exponentially with the size of the query graph, as noted above. Furthermore, the order in which the nodes are traversed is important. For large data graphs, a breadth-first search (BFS), in which all nodes at a given depth (level) are explored before moving onto the next level, would suffer large memory requirements. A DFS strategy is, therefore, more pragmatic, whereby each branch is explored to its end before backtracking and moving onto the next branch.

While the brute-force DFS strategy, denoted henceforth by bfDFS, circumvents large memory requirements, traversing the entire search tree is still impractical for all but the smallest data graphs. Established subgraph isomorphism algorithms aim to drastically reduce the size of the search space without introducing too much computational overhead. A straightforward refinement to bfDFS is to preemptively reject mappings in which the same data vertex is paired with more than one query vertex. The first, fifth, and ninth branches of the tree in Figure 3 would then be effectively discarded or “pruned”. In addition to this, one can check the feasibility of each partial trial mapping each time a new query–data vertex pair is introduced, rather than waiting until every query vertex is paired. If a partial (trial) mapping violates any of the subgraph isomorphism criteria, then it is abandoned, thus pruning the prevailing branch of the search tree.

Both of these “pruning rules” are exploited in the default pattern matching routines of *Zacros* 3.01, used for detecting both lattice processes and lateral interactions. A further refinement exploits the fact that only the patterns involving newly adsorbed product species must be detected following each event. Each trial mapping therefore starts with at least one query–data vertex pair already “locked in” at the root of the search tree. Following ref (42), we can say that the corresponding adsorbate is “fixed” on the pattern, with the number of “fixed” vertex pairs equal to the denticity of that adsorbate. Clearly, it is pointless to try to map lattice sites beyond a “local neighborhood”, the size of which depends on that of the pattern under consideration. More precisely, this “local neighborhood” can be found by computing the length *l* of the longest path that connects a fixed query vertex with a nonfixed one and finding all the data vertices that exist within *l* edges of the corresponding “fixed” data vertices (the latter being those mapped to the “fixed” query vertices). This way, one obtains the set  that essentially contains the (nonfixed) data vertices within *l* edges of the adsorbate. One can rule out matching any data vertices not in , and include only elements of  in the trial mappings, leading to a potentially drastic reduction in the size of the search tree. We refer to our “refined” DFS algorithm as rDFS, rather than “Ullmann’s algorithm” as in ref (41), so as to avoid confusion with the bit-matrix manipulations described in Ullmann’s original paper.^63^ However, the pruning rules involved in rDFS are inspired by those described therein.

#### More Advanced Depth-First Search Algorithms: VF2 and RI

While the rDFS algorithm has been used successfully to solve subgraph isomorphism problems in a range of GT-KMC simulations, the computational burden can be significant when the query graphs are large. This is relevant mostly for the detection of lateral interactions as this is usually the simulation bottleneck. To reduce the search space more aggressively, algorithms such as VF2 and RI start with a strategic choice of the order in which the query vertices will be matched, unlike rDFS in which the order is arbitrary. VF2 adopts a simple, dynamic ordering strategy, whereby one always searches for matches of the query vertex that has the highest degree (number of neighbors) among the neighbors of those previously matched.^68^ This way, query vertices with more stringent connectivity requirements are generally matched earlier. In contrast, RI adopts a static ordering strategy in which the sequence **μ** of query vertices is determined and stored before any matching takes place. This ordering strategy follows a similar principle as that of the VF2 algorithm but specifically tries to favor query vertices that are “more connected” to those already matched.^72^ This is achieved by assigning a lexicographic score to each query vertex *i* ∉ **μ**(*i* ∈ Φ) based on the cardinality of three different sets; the highest scoring vertex is then appended to **μ**. This process is iterated until **μ** includes all the query vertices. For a given query vertex *i* ∉ **μ** the three sets in order of priority are as follows:

1. *S*_vis_ is the set of vertices in **μ** that are neighbors of vertex *i*.
2. *S*_neig_ is the set of vertices in **μ** that are neighbors of at least one query vertex outside **μ** that is a neighbor of vertex *i*.
3. *S*_unv_ is the set of vertices that are neither in **μ** nor neighbors of vertices in **μ** but are neighbors of vertex *i*.

The ordering strategies just described provide any benefit only if they are combined with sensible choices for the domain of each query vertex *i*, denoted by domain(*i*). This is the set of data vertices to which one attempts to map query vertex *i*; in the case of rDFS, as we have seen, it includes (rather crudely) all of the unmatched vertices within *l* edges of the fixed adsorbates. In VF2, domain(*i*) includes just the unmatched neighbors of the previously matched data vertices.^68^ RI is stricter still, with domain(*i*) comprising just the unmatched neighbors of a *single* data vertex that has previously been matched with a neighbor of *i*. More precisely, while computing the ordered sequence of query vertices **μ**, we also determine the “parent” pt(*i*) of each vertex *i* ∈ **μ**, where pt(*i*) is defined as the first (i.e., lowest-index) member of **μ** that is a neighbor of *i*. Then, domain(*i*) consists of the unmatched neighbors of .^72^

Finally, while all of the isomorphism algorithms search for matches meeting the same criteria, the order and manner in which these criteria are checked can affect the execution speed. In rDFS and VF2, for each new trial pairing of *i* with , one first checks whether the connectivity satisfies Crit. 1, i.e., whether there exists a data edge  for each neighbor *j* of query vertex *i*. If so, Crit. 2 is checked by comparing the site types and coverages of query vertex *i* and data vertex , as well as any relevant geometric constraints. A different approach is taken in RI to account for the more aggressive connectivity-based pruning rules imposed by this algorithm. Thus, based on the assumption that relatively few trial mappings will fail to satisfy Crit. 1, Crit. 2 is checked first. Furthermore, Crit. 1 is only checked rigorously if  is first found to satisfy the weaker requirement that data vertex  has at least as many neighbors as query vertex *i*.

### Implementation in *Zacros*

#### Pattern Detection Routines

The original (“legacy”) implementation of rDFS included in *Zacros* 3.01 remains the default option for detecting both lattice processes (above a certain level of complexity; see below) and lateral interactions. We refer to that implementation as rDFS-lgy. An alternative “modern” implementation, rDFS-mdn, of the same algorithm is also included as a method of a stand-alone subgraph isomorphism class. While rDFS-lgy and rDFS-mdn are algorithmically equivalent, the former does not employ recursion; instead, it carries out an explicit loop over trial mappings of the nonfixed query vertices to members of . One may view this as iterating through partial permutations of . Each permutation is built up one site at a time such that invalid (“unfeasible”) partial mappings can be immediately rejected.

In contrast, rDFS-mdn is implemented as a recursive subroutine (Scheme S1 in the Supporting Information). The recursion is expected to carry some computational overhead, but the code structure lends itself to the development of more advanced algorithms more naturally than that of rDFS-lgy. Specifically, the “matching” part of the algorithm, in which the next query vertex and its domain are determined, is implemented as a separate method (Scheme S2), as is the “feasibility” check, which determines whether a given (partial) trial mapping satisfies Crit. 1 and 2 (Scheme S3). In fact, Crit. 2 is assessed by calling an external feasibility or “compatibility” function (Scheme S4), which is not part of the subgraph isomorphism module but rather of that handling the lattice state. Note that all the pseudocode presented in Schemes S1–S10, while written with lateral interactions in mind, is equally applicable to lattice process detection by substituting the relevant variables , etc.

Our VF2 implementation (Scheme S5) calls a unique matching function (Scheme S6) but the same feasibility function as rDFS-mdn. In contrast, our RI implementation (Scheme S7) calls both a unique matching function (Scheme S8) and a unique feasibility function (Scheme S9), which enables Crit. 2 to be checked before Crit. 1, as explained in the section Algorithms. Furthermore, the query vertices must be ordered in a preprocessing step, which is carried out by means of calling the RI_Order procedure (Scheme S10).

While lateral interactions are always detected using either rDFS-lgy or one of the recursive subgraph isomorphism procedures, these approaches are applied only to lattice processes when the mechanism (i.e., set of all elementary steps) exceeds a certain level of complexity. Specifically, when the mechanism involves only up to two-site steps and monodentate species, it is faster to follow a simpler approach. For each entity, the code identifies the species and loops over the elementary steps in which it can participate. In the case of a one-site step, provided the site type is correct, the corresponding lattice process is added to the queue. For a two-site step, a second loop is required to cycle over the neighbors of the first site.

#### Parallelization

Refs (33) and (42) detail how shared-memory (OpenMP) parallelization has already been used to accelerate GT-KMC simulations with *Zacros*. The most costly part of the simulation is the update of rate constants (and, in turn, future event times) following the execution of each event since this is where the detection of lateral interactions takes place. OpenMP parallelization was therefore implemented over the loop that cycles through the scheduled events within the neighborhood of the most recent lattice process. Thus, the new rate constants are calculated in parallel and stored in thread-private arrays.

We have extended the parallelization to work for simulations utilizing the new subgraph isomorphism class for lateral interaction detection. To achieve this, a thread-private instance of this class must be created for each thread. We also create thread-private instances of the class that handles the data required for the external feasibility check.

## Results and Discussion

### NO Oxidation on Pt(111)

Our first set of benchmarks is performed on the NO oxidation model established by Schneider and co-workers^32,61^ and adapted within the KMC framework by Nielsen et al.^33^ This model considers atomic oxygen on Pt(111) as the only adsorbate in the system, denoted as O*, and assumes that adsorbed oxygen and gaseous NO are in quasi-equilibrium with gaseous NO_2_; thus, the dissociation of gaseous O_2_ is the rate-limiting step. Underpinning this model is a hierarchy of CEs for O^*^ developed by Schmidt et al.,^61^ who used DFT calculations to fit progressively larger CEs, capturing longer ranges of lateral interactions. Schmidt et al. thus found that a 12-figure CE, including up to eighth nearest-neighbor pairwise interactions and two triplets (three-body interactions), is sufficient to predict formation energies and ground states accurately.^61^ Wu et al.^32^ then employed this model in a study that combined equilibrium MC with a microscopic rate-averaging scheme to predict the apparent rate of catalytic NO oxidation on Pt(111).

Based on the CEs of Schmidt et al.^61^ and the kinetic parameters calculated by Wu et al.,^32^ Nielsen et al.^33^ built a corresponding set of GT-KMC models for the same NO oxidation reaction. Specifically, they benchmarked an earlier version of *Zacros* in which the detection of lateral interactions was already parallelized but limited to what we now call rDFS-lgy. They found that the execution was more than 4 orders of magnitude slower when using the most complex energetic model (12-figure) compared to the simplest (3-figure), illustrating the need to reduce the computational expense of computing long-range lateral interactions. Parallelizing the rate constant updates using OpenMP was shown to be highly effective for the 12-figure model, with execution speed scaling almost linearly. In contrast, parallelization with the 3-figure model was found to yield only modest speedups, plateauing at around 2× as the number of threads approaches 16. This was explained by the increasing time required to synchronize the threads when collecting the updated rate constants, which eventually becomes the simulation bottleneck when the number of rate constants affected by each event is small (i.e., the lateral interactions are short-range).

We have employed the same NO/Pt(111) reaction model and set of CEs to assess the improvements in performance yielded by our “modern” pattern detection routines. As described in ref (33), NO oxidation proceeds via the following reversible elementary steps
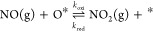
17a
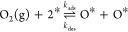
17b
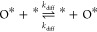
17c

**Figure 4:**
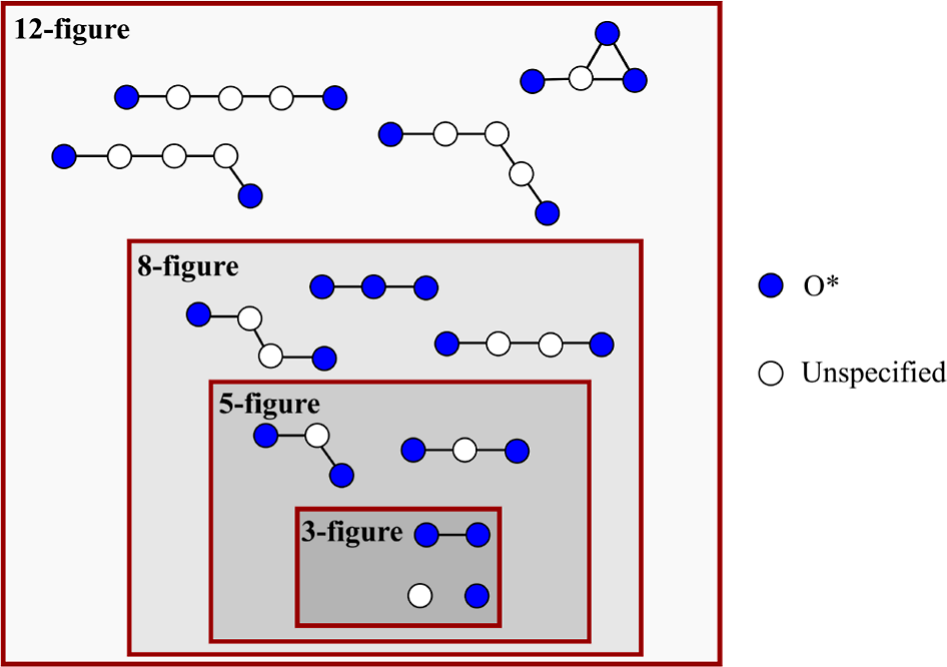
Energetic interaction patterns included in Schmidt et al.’s hierarchy of cluster expansions (CEs) for O* on Pt(111).^61^ The underlying lattice is omitted for simplicity; for more detail, see Figure 2 of ref (33). We use blue filled circles to represent lattice sites occupied by O^*^ and white filled circles to represent “nonspecific sites” (i.e., sites with an “unspecified” state), which may participate in the pattern regardless of their occupancy (see the section Adsorbate Lateral Interactions).

These constitute NO oxidation, O_2_ dissociative adsorption, and O* diffusion, respectively. Details of the graph representations and kinetic parameters can be found in ref (33). The energetic models and corresponding graph patterns are also described in detail in ref (33), but to guide our discussion, in Figure 4, we provide a qualitative illustration of the figures included in each CE. The 3-figure expansion includes only point (1-body) terms and the first nearest-neighbor (1NN) effective O* – O* interaction, while the 5-, 8-, and 12-figure expansions cover up to 3NN, 5NN, and 8NN terms, respectively. The 8- and 12-figure expansions also include some triplet terms.

Our simulations employed a lattice size of 42 × 42 (1764 sites), with the temperature fixed at 480 K, total pressure at 1 bar, and the molar fractions of the reactive gas species at *y*_O_2__ = 0.1, *y*_NO_ = 4.139938 × 10^–9^, and *y*_NO_2__ = 1.522998 × 10^–9^. Starting from a steady-state adlayer structure, we ran a series of simulations using each combination of CE and pattern detection algorithm, with the rate constant updates parallelized over different numbers of threads. Each simulation was terminated after 1 h of clock time had elapsed. All simulations were carried out on type D nodes of the computational cluster Myriad@UCL, each containing 36 cores and 192 GB of RAM. We obtained two sets of results to compare the GNU and Intel Fortran compilers. Our performance metric is the acceleration factor, defined as the rate of event execution relative to that for a single-threaded run using rDFS-lgy for lateral interaction detection. Note that distinct from a “serial” run, a “single-threaded” run employs the OpenMP-enabled *Zacros* executable with the environment variable OMP_NUM_THREADS set to 1. It is also worth highlighting that the reaction mechanism involves only one- and two-site patterns and monodentate adsorbates, so lattice process detection is always carried out using the simpler method for elementary event detection described in Implementation in *Zacros*. This is not a simulation bottleneck.

**Figure 5:**
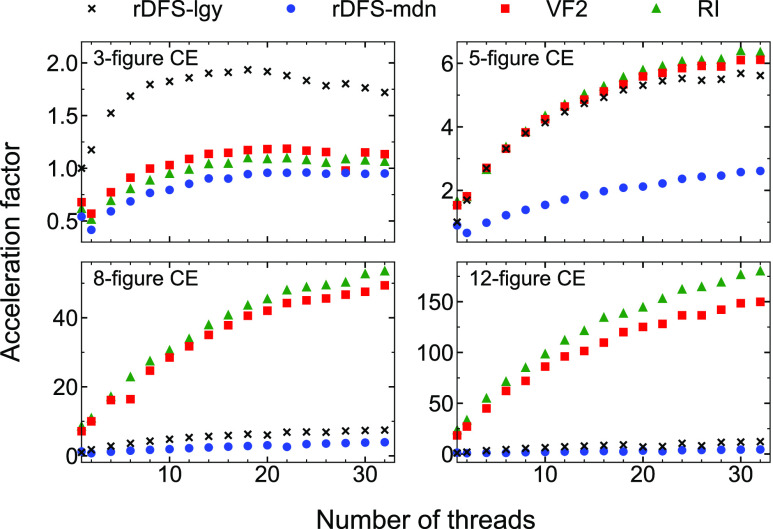
Plots of acceleration factors as a function of the number of threads for GT-KMC simulations of NO oxidation on Pt(111). The acceleration factor is defined with respect to a single-threaded run using rDFS-lgy to detect lateral interactions. These results were obtained using *Zacros* compiled with GNU Fortran.

**Figure 6:**
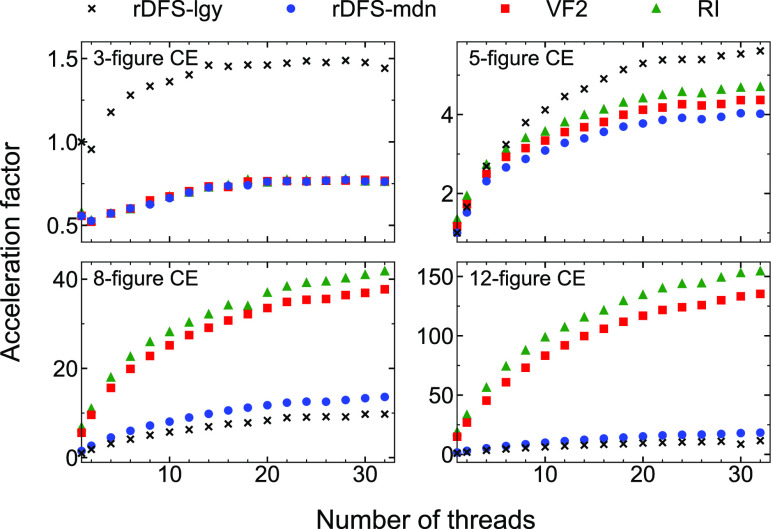
As in Figure 5 but obtained using *Zacros* compiled with Intel Fortran.

The GNU and Intel acceleration factors are plotted with respect to the number of threads (processors) in Figures 5 and 6, respectively. For the 3-figure CE, as discovered by Nielsen et al.^33^ and noted above, parallelization provides only modest speedups relative to single-threaded runs. The maximum acceleration factor is greater for the GNU-compiled version of *Zacros* than for the Intel-compiled version, although, interestingly, this maximum is reached at 16 threads, beyond which the event execution starts to slow down. Using any of the “modern” pattern detection routines also slows down the simulation. The latter is indicative of computational overheads involved in recursion and in initializing and manipulating the data structures needed to represent each mapping . The comparable performances of rDFS-mdn, VF2, and RI suggest that the “aggressive” approaches to search space reduction adopted in VF2 and RI are, in this case, excessive. This point is explored further below.

For the 5-figure CE, VF2 and RI display similar performance to rDFS-lgy. With the GNU version, rDFS-mdn is considerably slower and parallelizes less efficiently. In the Supporting Information, we show that this effect is somewhat lattice size-dependent as for large lattices, data duplication among threads becomes a bottleneck. Interestingly, with Intel-compiled *Zacros*, rDFS-mdn performs almost as well as the other algorithms. Overall, our results suggest that VF2 and RI offer some algorithmic advantage in terms of reducing the search space, but this is only just able to compensate for the overhead of recursion.

In stark contrast, simulations run with the 8- and 12-figure CEs can be sped up drastically by using either VF2 or RI to detect lateral interactions. Taking the Intel results as an example, single-threaded runs with VF2 progress around 7 and 18 times faster than those using rDFS-lgy for the 8- and 12-figure CEs, respectively. The RI algorithm proves to be marginally faster still, with acceleration factors of roughly 9 and 24. Combining RI with parallelization over 32 processors, the acceleration factors reach 42 (8-figure) and 154 (12-figure). They are even more impressive with GNU Fortran compilation, reaching 53 (8-figure) and 180 (12-figure).

It is pertinent to ask why the VF2 and RI algorithms provide such large speedups relative to rDFS with the larger CEs but none at all with the smallest CE. To address this question, we examined the effectiveness of the search space reduction performed by each algorithm, keeping in mind that the pruning rules are based solely on connectivity and thus do nothing to eliminate partial matches violating Crit. 2 (compatibility). Accordingly, we define the “partial match success rate” (PMSR) to be the fraction of attempted partial matches found to satisfy Crit. 1. The ideal scenario, in which the search tree contains the smallest possible number of branches, is represented by a PMSR of 1.00, i.e., when every partial match attempted has acceptable connectivity.

Table 1 gives the PMSRs of lateral interaction detection for a series of 1 h, single-threaded simulations of NO oxidation on Pt(111). All three algorithms display “ideal” behavior for the 3-figure CE; hence, as Figures 5 and 6 revealed, there is no advantage to the intricate ordering strategies and pruning rules of VF2 or RI relative to the simplistic approach of rDFS. Additionally, larger figures are added to the CE and the PMSR of rDFS drops drastically, with only 5% of attempted partial matches satisfying Crit. 1 in the 12-figure case. In contrast, the PMSR of VF2 drops much more gradually, reaching a minimum of 0.46, which is consistent with Figures 5 and 6 and indicates that the search space reduction is fairly effective, even for large query graphs. Remarkably, the PMSR of RI remains at 1.00 up to and including the 8-figure CE, dropping only slightly to 0.89 for the 12-figure CE. RI is therefore almost as efficient as any algorithm can be for detecting lateral interactions in NO/Pt(111), insofar as one is concerned with minimizing the size of the search tree.

**Table 1:** Partial Match Success Rates

size of CE	PMSR		
	rDFS	VF2	RI
3	1.00	1.00	1.00
5	0.30	0.68	1.00
8	0.08	0.61	1.00
12	0.05	0.46	0.89

In practice, Table 1 only tells part of the story. For the 12-figure expansion, while RI is almost twice as effective as VF2 at eliminating partial matches that violate Crit. 1, the two algorithms yield similar KMC execution speeds. This is because much of the execution time is spent on checking whether Crit. 2 is satisfied, which involves comparing the site types, occupancies, and geometric properties of cluster and lattice vertices. As explained in More Advanced Depth-First Search Algorithms: VF2 and RI, RI performs this time-consuming step before checking Crit. 1, whereas rDFS and VF2 check Crit. 1 first. Therefore, since ∼90% of partial matches in RI satisfy Crit. 1 (see Table 1), we know that VF2 needs to check Crit. 2 ∼90% as many times as RI. Profiling with Arm MAP^86^ revealed that for a single-threaded *Zacros* run, ∼60% of the total time is spent on checking Crit. 2 when RI is used to detect lateral interactions. In contrast, when VF2 is used, checking Crit. 2 accounts for ∼40% of the total time.

### Methane Cracking on Ni(111)

As a more realistic demonstration of the improvement in performance yielded by our modern pattern detection routines, we used *Zacros* to carry out KMC simulations of methane cracking on Ni(111). The reaction model was developed by Yadavalli et al.,^76^ with a complex mechanism involving 2 site types, 5 adsorbate species, and 10 reversible elementary steps. These include all of the C–H activation steps leading from CH_4_ up to C + 4H, such that the kinetics of dehydrogenation are captured in detail, whereas the formation of coke is captured at the level of thermodynamics only. A 62-figure CE is used to capture the effective cluster interactions between adsorbed CH_*x*_ species (*x* = 0, ..., 3) and covalently bonded C and CH species. This comprises pairwise interaction parameters up to the 3NN level, as well as several higher-level clusters, with the largest figure in the CE containing 5 sites. For full details of the mechanism and energetics model, the reader is referred to ref (76).

We set out to compare the performance of rDFS-lgy with that of RI, in both cases parallelizing the rate constant updates over 32 processors. Simulations were initialized with an empty lattice, a temperature of 1000 K and a pressure of 10.01 bar. To shed light on how surface coverage affects the execution speed, we stopped and restarted each simulation at KMC time intervals of 0.2 ms, up to a final KMC time of 5 ms. Thus, as a dynamic performance metric, we could estimate the rate of clock time advancement with respect to KMC time
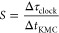
18where τ_clock_ and *t*_KMC_ denote the clock time and KMC time, respectively. Note that *better* performance (i.e., faster KMC execution) is characterized by a *smaller* value of *S*. As for the NO oxidation system, all methane cracking simulations were carried out on type D nodes of the computational cluster Myriad@UCL. The compiler used was GNU Fortran.

**Figure 7:**
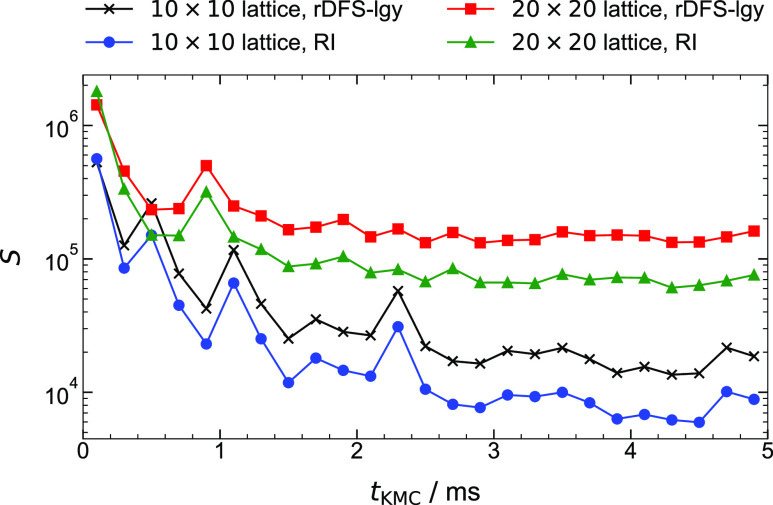
Results of GT-KMC performance benchmarks of the methane cracking model introduced in ref (76). We plot *S* against *t*_KMC_, where *S* is the clock time advanced per unit of KMC time (see eq 18) and *t*_KMC_ is the KMC time. These results were obtained using *Zacros* compiled with GNU Fortran.

In Figure 7, we plot *S* as a function of *t*_KMC_ for the methane cracking model with two different lattice sizes, 10 × 10 and 20 × 20. The results for each lattice size have been averaged over 5 repetitions of the simulation (all using identical pseudorandom number streams). Notwithstanding some fluctuation, it is clear that *S* generally decreases as the simulation progresses, meaning that the KMC time advances more quickly. This is because when the lattice is mostly empty, the process queue contains a large number of adsorption events, each of which increments the KMC time by a relatively small amount. In contrast, as the system approaches steady-state behavior, the surface becomes “poisoned” by carbonaceous species, resulting in slower dynamics and, correspondingly, large interarrival times.

Turning our attention to the method of detecting lateral interactions, rDFS-lgy and RI show similar performance in the early stages of the simulation. This is easy to explain: on a surface with low coverage, the adlayer is almost ideal; thus, there are few interaction patterns to detect. As the CH/C species accumulate on the surface (comprising mostly ring-based structures), long-range interactions between them start to emerge; thus, the fraction of computational time dedicated to solving subgraph isomorphism problems increases. Accordingly, we see that the speedup offered by RI becomes more significant, exceeding 2× at *t*_KMC_ = 5 ms. Thus, the long time scales involved in the surface poisoning process have become more readily accessible, simplifying the elucidation of the “terminal” state of coking.^76^

## Conclusions

In the graph-theoretical kinetic Monte Carlo (GT-KMC) framework, which is implemented in the *Zacros* software package, adsorbate lateral interactions are described by using the cluster expansion (CE) formalism. There, the formation energy of the adlayer configuration is expanded as a series of effective “cluster” interaction energies. To accurately compute the activation energies of elementary lattice processes, one then has to identify mappings between the clusters/figures of the CE and patterns of sites on the lattice. This amounts to solving a series of subgraph isomorphism problems and is analogous to the way the lattice processes are themselves identified.^33,41^

When the lateral interactions span long distances, the associated pattern detection becomes computationally highly demanding. Previous work to reduce this demand has focused on developing schemes for efficient parallel processing^33^ and caching,^42^ with fairly promising results. Here, we have built on this work by directly addressing the algorithms used to identify subgraph isomorphisms. The original choice of algorithm for *Zacros*, termed rDFS, adopts a fairly crude approach to reducing the size of the search space that is not always effective. We implemented two more sophisticated algorithms, namely, VF2 and RI, alongside rDFS in a “family” of recursive procedures. The last of these procedures is termed rDFS-mdn for the sake of distinguishability from the “legacy” implementation rDFS-lgy of the same algorithm, which remains in the code as well.

We benchmarked the performance of our “modern” pattern detection routines on a hierarchy of models of catalytic NO oxidation on Pt(111), developed in refs (32), (33), (61). These models treat the interactions between adsorbed oxygen atoms with varying levels of accuracy. The simplest of the models includes just 3 figures in the CE Hamiltonian, incorporating only up to first nearest-neighbor (1NN) pairwise interactions, while the most complex model includes 12 figures, incorporating up to 8NN pairwise interactions alongside some three-body terms. We found that VF2 and RI provide large improvements in performance with the more complex energetic models, yielding acceleration factors up to ∼180 (compared to single-thread runs) when combined with shared-memory parallel processing. On the other hand, rDFS-lgy is the best option when one only needs to detect short-range patterns, for which a simple search-space reduction strategy is adequate.

As a more realistic test, we compared the performance of RI to that of rDFS-lgy for computing lateral interactions in simulations of methane cracking on Ni(111).^76^ At low surface coverage, when the number of adsorbate molecules interacting is small, the difference in performance was found to be small as well. However, as the lattice approached a poisoned state, the simulation using RI progressed more than twice as fast.

We conclude that KMC simulations of catalytic surface phenomena may be considerably accelerated by using sophisticated subgraph isomorphism algorithms for the detection of lateral interactions. Further, the acceleration is most significant when the adlayer contains a large number of long-range patterns. We expect that our implementation of VF2 and RI in *Zacros* will advance catalyst discovery by enabling simulations involving complex energetic models that were previously considered intractable.

## Supporting Information Available

Pseudocode for the subgraph isomorphism algorithms implemented in *Zacros*, and additional GT-KMC benchmarks of the NO/Pt(111) system with the 5-figure cluster expansion comparing the performances of different algorithms across a range of lattice sizes (PDF)
