# Supplementary material for: Speeding up the Detection of Adsorbate Lateral Interactions in Graph-Theoretical Kinetic Monte Carlo Simulations
Source: J Phys Chem A. 2023 Nov 21;127(48):10307–19. doi: 10.1021/acs.jpca.3c05581 (PMC11065322; doi:10.1021/acs.jpca.3c05581)
Supplement: Supplementary file 1 — jp3c05581_si_001.pdf [file jp3c05581_si_001.pdf]

Speeding Up the Detection of Adsorbate Lateral Interactions in Graph-Theoretical Kinetic Monte Carlo Simulations

Raz L. Benson,[†] Sai Sharath Yadavalli,[†] and Michail Stamatakis,[*,†,‡]

[†] Department of Chemical Engineering, University College London, Torrington place, London, WC1E 7JE, United Kingdom.

[‡] Current address: Inorganic Chemistry Laboratory, University of Oxford, S Parks Rd, Oxford, OX1 3QR, United Kingdom

[*] E-mail: michail.stamatakis@chem.ox.ac.uk

SUPPORTING INFORMATION

S1 Pseudocode for subgraph isomorphism algorithms

Scheme S1: Refined depth-first search procedure for detecting lateral interaction patterns

Procedure: **`rDFS_Recursive`**$(\mathcal{M})$
Data: Set of isomorphisms $\mathcal{I}$ already found, initially $\varnothing$
Input: Verified partial mapping $\mathcal{M}$, which initially covers only fixed sites
Result: Compute all required isomorphisms and store in $\mathcal{I}$
begin
  if $\mathcal{M}$ covers all query vertices **then**
    $\mathcal{I} \longleftarrow \mathcal{I} \cup \{\mathcal{M}\}$
  else
    i, domain$(i) \longleftarrow$ `rDFS_Match`$(\mathcal{M})$
    for $j \in$ domain(i) **do**
      `// If query vertex` i `is specific, we must attempt to map it to each of the data vertices in its domain. Otherwise, we iterate only as far as required to yield one complete isomorphism. rDFS_Match (Scheme S2) ensures that` i `will only be non-specific once` $\mathcal{M}$ `already covers all specific query vertices.`
      if $\chi_i \neq \&$ (i.e., query vertex i is 'specific') **or** $\mathcal{I}$ contains no isomorphisms with the same specific vertex mappings as $\mathcal{M}$ **then**
        $\mathcal{M}(i) \longleftarrow j$
        if `rDFS_Feasibility`$(\mathcal{M}, i)$ **then call** `rDFS_Recursive`$(\mathcal{M})$
        $\mathcal{M}(i) \longleftarrow$ `null`

Scheme S2: Determine the next query vertex to match in the rDFS procedure and its domain

Function: `rDFS_Match`$(\mathcal{M})$
Data: Set of non-fixed data vertices $\mathcal{S}_{\mathrm{loc}}$ within l edges of the fixed data vertices
Set Φ of query (cluster) vertices
Vectors $\chi_{i'}, i' \in \Phi$ representing cluster coverage pattern
Input: Verified partial mapping $\mathcal{M}$
Output: Index i of the next query vertex to match
Set of data vertices domain(i) to which to attempt to map query vertex i
begin
  domain$(i) \longleftarrow \varnothing$
  for $j \in \mathcal{S}_{\mathrm{loc}}$ **do**
    if $\mathcal{M}$ does not map any query vertices to j **then**
      domain$(i) \longleftarrow$ domain$(i) \cup \{j\}$
  if $\mathcal{M}$ covers all specific query vertices **then**
    for $i' \in \Phi$ **do**
      if $\mathcal{M}(i') =$ `null` **and** $\chi_{i'} = \&$ **then**
        $i \longleftarrow i'$
        return i, domain(i)
  else
    for $i' \in \Phi$ **do**
      if $\mathcal{M}(i') =$ `null` **and** $\chi_{i'} \neq \&$ **then**
        $i \longleftarrow i'$
        return i, domain(i)

Scheme S3: Determine whether a trial partial mapping in the rDFS procedure is feasible

Function: `rDFS_Feasibility`$(\mathcal{M}, i)$
Data: Set Φ of query (cluster) vertices
Set $\mathcal{S}$ of data (lattice) vertices
Query graph adjacency lists neigh$_{\mathcal{C}}(i') = \{i'' | (i', i'') \in \Pi\}, i' \in \Phi$
Data graph adjacency lists neigh$_{\mathcal{L}}(j) = \{j' | (j, j') \in \mathcal{E}\}, j \in \mathcal{S}$
Input: Trial partial mapping $\mathcal{M}$
Index i of the query vertex whose mapping validity is to be checked
Output: `true` if $\mathcal{M}$ is a valid partial mapping, `false` otherwise
begin
  `// Internal feasibility check (Crit. 1)`
  for $j \in$ neigh$_{\mathcal{C}}(i)$ **do**
    if $\mathcal{M}(j) \neq$ `null` **and** $\mathcal{M}(j) \notin$ neigh$_{\mathcal{L}}\big(\mathcal{M}(i)\big)$ **then return** `false`
  `// External feasibility check (Crit. 2)`
  if `isCompatible`$(\mathcal{M}, i)$ **then**
    return `true`
  else
    return `false`

Scheme S4: Determine whether a trial partial mapping satisfies Crit. 2

Function: `isCompatible`$(\mathcal{M}, i)$

Data: Set Φ of query (cluster) vertices
Set $\mathcal{S}$ of data (lattice) vertices
Cluster site types $\phi_{i'}, i' \in \Phi$
Lattice site vectors, $\mathbf{s}_{i'}, i' \in \mathcal{S}$
Cluster state vectors $\chi_{i'}, i' \in \Phi$
Lattice state vectors $\boldsymbol{\sigma}_{i'}, i' \in \mathcal{S}$
Cluster geometric constraints of the form $\text{Angle}(\mathcal{C}; i, j, k) = \varphi_{ijk}, i, j, k \in \Phi$
Angle tolerance $\Delta\varphi$
List of excluded adsorbates (lattice entities) associated with each cluster entity that is fixed on the pattern (needed to avoid double counting new lateral interaction patterns—see ref. 1)

Input: Trial partial mapping $\mathcal{M}$
Index i of the query vertex whose mapping validity is to be checked

Output: `true` if $\mathcal{M}$ satisfies Crit. 2, `false` otherwise

begin
  `// Check site types (Crit. 2(a))`
  if $\phi_i \notin \{s_{\mathcal{M}(i),1}, 0\}$ **then return** `false`
  `// Check states (Crit. 2(b))`
  if query vertex i is specific **then**
    if $\sigma_{\mathcal{M}(i),2} \neq \chi_{i,2}$ **then return** `false`
    if $\sigma_{\mathcal{M}(i),3} \neq \chi_{i,3}$ **then return** `false`
  Set $\mathcal{F}(k)$ to `null` for all cluster entities $k \in \{1, \ldots, \sum_{j\in\Phi} 1/d_{\chi_{i,2}}\}$
  for $j \in \Phi$ **do**
    if $\mathcal{M}(j) \neq$ `null` **and** query vertex j is specific **then**
      if $\mathcal{F}(\chi_{j,1}) =$ `null` **then**
        $\mathcal{F}(\chi_{j,1}) \longleftarrow \sigma_{\mathcal{M}(j),1}$
        `// Reject any patterns in which an ‘excluded adsorbate’ participates with entity number less than that of the corresponding fixed cluster entity (see ref. 1 Scheme 1, Fig. 2 and text on pg. 7144)`
        for each entity k that is fixed on the pattern **do**
          if $\chi_{j,1} < k$ **and** $\mathcal{F}(\chi_{j,1})$ is in the excluded adsorbates list associated with entity k **then**
            return `false`
      else if $\mathcal{F}(\chi_{j,1}) \neq \sigma_{\mathcal{M}(j),1}$ **then**
        return `false`
  `// Check geometry (Crit. 2(c))`
  for each angle specified as $\text{Angle}(\mathcal{C}; i, j, k) = \varphi_{ijk}, i, j, k \in \Phi$ **do**
    if $|\text{Angle}(\mathcal{L}; \mathcal{M}(i), \mathcal{M}(j), \mathcal{M}(k)) - \varphi_{ijk}| > \Delta\varphi$ **then return** `false`
  return `true`

Scheme S5: VF2 procedure for detecting lateral interaction patterns

Procedure: `VF2_Recursive`($\mathcal{M}$)
Data: Set of isomorphisms $\mathcal{I}$ already found, initially $\emptyset$
Input: Verified partial mapping $\mathcal{M}$, which initially covers only fixed sites
Result: Compute all required isomorphisms and store in $\mathcal{I}$
begin
  if $\mathcal{M}$ covers all query vertices **and** $\mathcal{I}$ contains no isomorphisms with the same specific vertex mappings as $\mathcal{M}$ **then**
    $\mathcal{I} \longleftarrow \mathcal{I} \cup \{\mathcal{M}\}$
  else
    i, domain(i) $\longleftarrow$ `VF2_Match`($\mathcal{M}$)
    for $j \in$ domain(i) **do**
      $\mathcal{M}(i) \longleftarrow j$
      if `rDFS_Feasibility`($\mathcal{M}, i$) **then call** `VF2_Recursive`($\mathcal{M}$)
      $\mathcal{M}(i) \longleftarrow$ `null`

Scheme S6: Determine the next query vertex to match in the VF2 procedure and its domain

Function: `VF2_Match`($\mathcal{M}$)
Data: Query graph adjacency lists $\text{neigh}_\mathcal{C}(i') = \{i'' | (i', i'') \in \Pi\}, i' \in \Phi$
Data graph adjacency lists $\text{neigh}_\mathcal{L}(j) = \{j' | (j, j') \in \mathcal{E}\}, j \in \mathcal{S}$
Query vertex degrees $\text{deg}_\mathcal{C}(i') = |\text{neigh}_\mathcal{C}(i')|, i' \in \Phi$
Input: Verified partial mapping $\mathcal{M}$
Output: Index i of the next query vertex to match
Set of data vertices domain(i) to which to attempt to map query vertex i
begin
  domain(i) $\longleftarrow \emptyset$
  MaxDeg $\longleftarrow 0$
  for $i' \in \Phi$ **do**
    if $\mathcal{M}(i') \neq$ `null` **then**
      for $j \in \text{neigh}_\mathcal{L}(\mathcal{M}(i'))$ **do**
        if $\mathcal{M}$ does not map any query vertices to j **and** $j \notin$ domain(i) **then**
          domain(i) $\longleftarrow$ domain(i) $\cup \{j\}$
      for $i'' \in \text{neigh}_\mathcal{C}(i')$ **do**
        if $\mathcal{M}(i'') =$ `null` **and** $\text{deg}_\mathcal{C}(i'') >$ MaxDeg **then**
          MaxDeg $\longleftarrow \text{deg}_\mathcal{C}(i'')$
          $i \longleftarrow i''$
  return i, domain(i)

Scheme S7: RI procedure for detecting lateral interaction patterns

Procedure: **RI_Recursive**($\mathcal{M}$)
Data: Set of isomorphisms $\mathcal{I}$ already found, initially $\varnothing$
Input: Verified partial mapping $\mathcal{M}$, which initially covers only fixed sites
Result: Compute all required isomorphisms and store in $\mathcal{I}$
begin
  if $\mathcal{M}$ covers all query vertices **and** $\mathcal{I}$ contains no isomorphisms with the same specific vertex mappings as $\mathcal{M}$ **then**
    $\mathcal{I} \longleftarrow \mathcal{I} \cup \{\mathcal{M}\}$
  else
    i, domain(i) $\longleftarrow$ RI_Match($\mathcal{M}$)
    for $j \in$ domain(i) **do**
      $\mathcal{M}(i) \longleftarrow j$
      if RI_Feasibility($\mathcal{M}, i$) **then call** RI_Recursive($\mathcal{M}$)
      $\mathcal{M}(i) \longleftarrow$ null

Scheme S8: Determine the next query vertex to match in the RI procedure and its domain

Function: **RI_Match**($\mathcal{M}$)
Data: Ordered sequence of query vertices $\boldsymbol{\mu}$
Parents vertices pt(i), $i \in \boldsymbol{\mu}$
Data graph adjacency lists $\text{neigh}_{\mathcal{L}}(j) = \{j' | (j, j') \in \mathcal{E}\}, j \in \mathcal{S}$
Input: Verified partial mapping $\mathcal{M}$
Output: Index i of the next query vertex to match
Set of data vertices domain(i) to which to attempt to map query vertex i
begin
  Set i to the next unmapped element of $\boldsymbol{\mu}$
  domain(i) $\longleftarrow \varnothing$
  for $j \in \text{neigh}_{\mathcal{L}}(\mathcal{M}(\text{pt}(i)))$ **do**
    if $\mathcal{M}$ does not map any query vertices to j **then**
      domain(i) $\longleftarrow$ domain(i) $\cup \{j\}$
  return i, domain(i)

Scheme S9: Determine whether a trial partial mapping in the RI procedure is feasible

Function: **RI_Feasibility**$(\mathcal{M}, i)$
Data: Query graph adjacency lists $\text{neigh}_{\mathcal{C}}(i') = \{i'' | (i', i'') \in \Pi\}, i' \in \Phi$
Data graph adjacency lists $\text{neigh}_{\mathcal{L}}(j) = \{j' | (j, j') \in \mathcal{E}\}, j \in \mathcal{S}$
Query vertex degrees $\text{deg}_{\mathcal{C}}(i') = |\text{neigh}_{\mathcal{C}}(i')|, i' \in \Phi$
Data vertex degrees $\text{deg}_{\mathcal{L}}(j) = |\text{neigh}_{\mathcal{L}}(j)|, j \in \mathcal{S}$
Input: Trial partial mapping $\mathcal{M}$
Index i of the query vertex whose mapping validity is to be checked
Output: `true` if $\mathcal{M}$ is a valid partial mapping, `false` otherwise
begin
    `// External feasibility check (Crit. 2)`
    if not `isCompatible`$(\mathcal{M}, i)$ **then return** `false`
    `// High-level internal feasibility check (Crit. 1)`
    if $\text{deg}_{\mathcal{L}}(\mathcal{M}(i)) < \text{deg}_{\mathcal{C}}(i)$ **then return** `false`
    `// Low-level internal feasibility check (Crit. 1)`
    for $j \in \text{neigh}_{\mathcal{C}}(i)$ **do**
        if $\mathcal{M}(j) \neq$ `null` **and** $\mathcal{M}(j) \notin \text{neigh}_{\mathcal{L}}(\mathcal{M}(i))$ **then return** `false`
    return `true`

Scheme S10: Determine order in which query vertices will be matched in the RI procedure

1 **Procedure:** **RI_Order**()
Result: Compute and store ordered sequence of query vertices $\boldsymbol{\mu}$
Compute and store query vertex 'parents' $\text{pt}(i), i \in \boldsymbol{\mu}$
2 **begin**
3   rank $\longleftarrow (-1, -1, -1)$
4     $m = 0$
5   Set i_m to the index of the fixed query vertex of maximum degree
6     $\boldsymbol{\mu} \longleftarrow (i_m)$
7   **while** $m <$ number of fixed query vertices **do**
8   **for** $i \in \Phi$ **do**
9   **if** site i is fixed on the pattern **and** $i \notin \boldsymbol{\mu}$ **then**
10   Compute $S_{\text{vis}} = \{i' | i' \in \boldsymbol{\mu}, (i_m, i') \in \Pi\}$
11   Compute $S_{\text{neig}} = \{i' | i' \in \boldsymbol{\mu}, \exists i'' \notin \boldsymbol{\mu} | (i', i'') \in \Pi, (i_m, i'') \in \Pi\}$
12   Compute $S_{\text{unv}} = \{i' | i' \notin \boldsymbol{\mu}, (i_m, i') \in \Pi, \forall i'' \in \boldsymbol{\mu}\ (i', i'') \notin \Pi\}$
13   **if** rank $\leq (|S_{\text{vis}}|, |S_{\text{neig}}|, |S_{\text{unv}}|)$ **then**
14                     $i_{m+1} = i$
15   rank $\longleftarrow (|S_{\text{vis}}|, |S_{\text{neig}}|, |S_{\text{unv}}|)$
16   Set $\text{pt}(i_{m+1})$ to `null`
17   Append i_{m+1} to $\boldsymbol{\mu}$
18         $m \longleftarrow m + 1$
19   **while** $m < N_{\Phi}$ **do**
20   **repeat** lines 8–15
21   Set $\text{pt}(i_{m+1})$ to first member of $\boldsymbol{\mu}$ such that $\text{pt}(i_{m+1}) \in \Pi$
22   Append i_{m+1} to $\boldsymbol{\mu}$
23         $m \longleftarrow m + 1$

S2 Lattice-size dependence of parallel efficiency

In the manuscript we reported on improvements in performance yielded by employing 'modern' subgraph isomorphism algorithms in tandem with shared-memory parallelisation for detecting lateral interaction patterns, focusing mostly on a model of NO oxidation on Pt(111) with a hierarchy of cluster expansions (CEs). For the 5-figure CE, when using *Zacros* compiled with GNU Fortran, rDFS-mdn was found to be significantly slower than the other algorithms and also to parallelise less efficiently. Here, we investigate whether this behaviour is affected by increasing the size of the lattice. To this end, we ran a series simulations with the 5-figure CE, similar to those described in the manuscript but employing a range of lattice sizes: 42×42, 210×210, 420×420 and 840×840. To obtain a sensible initial adlayer structure for each lattice size (i.e., one that approximates the steady state), the original 42×42 cell was tessellated (tiled) $n \times n$ times as appropriate, e.g. $n = 5$ for lattice size 210×210. Furthermore, rather than running each simulation for a fixed wall time, we fixed the number of events at $5000n^2$. Finally, it should be noted that these simulations were carried out on different hardware, namely a local workstation containing 32 physical cores (AMD Ryzen Threadripper 3970X) and 256 GB of RAM.

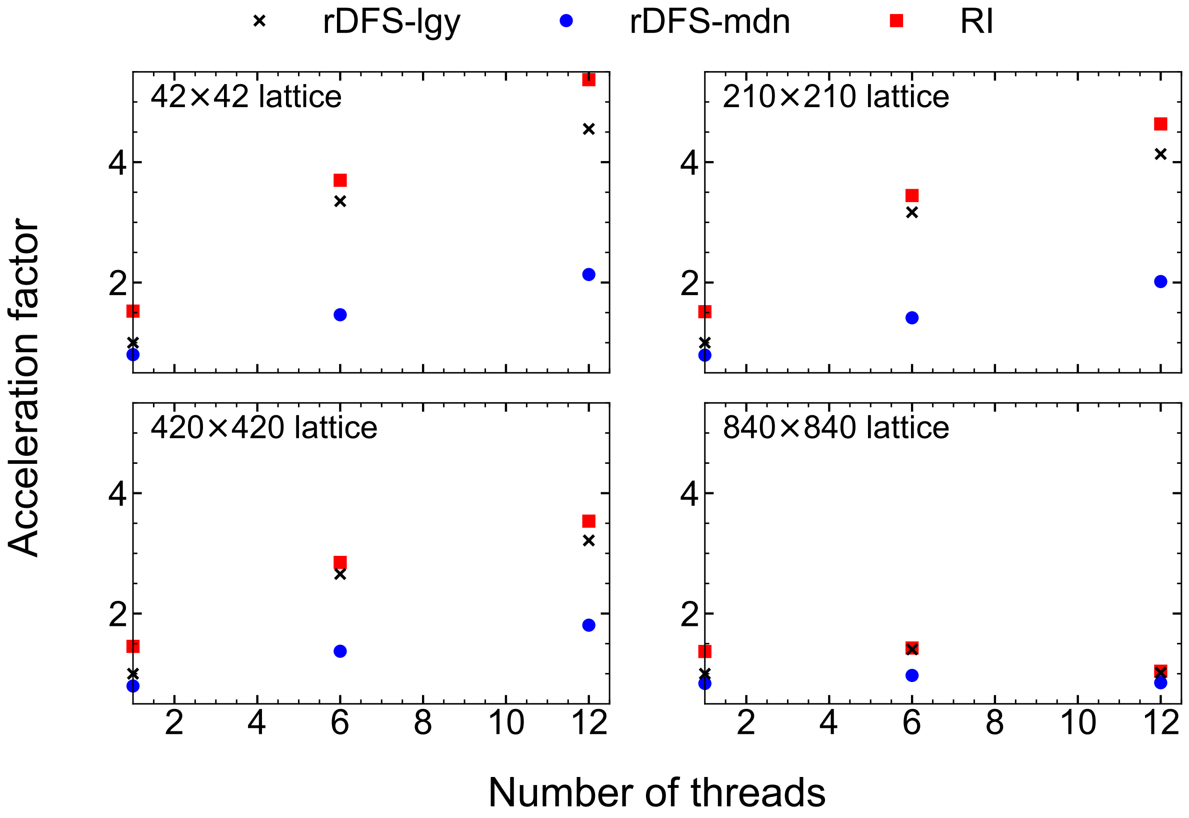

Figure S1. Plots of acceleration factor versus number of threads for GT-KMC simulations of NO oxidation on Pt(111), using a 5-figure cluster expansion with a range of lattice sizes. The acceleration factor is defined with respect to a single-threaded run using rDFS-lgy to detect lateral interactions. These results were obtained using *Zacros* compiled with GNU Fortran.

In Figure S1, we compare the acceleration factors achieved by rDFS-lgy, rDFS-mdn and RI for the different lattice sizes. As the lattice size increases from 42×42 to 420×420, the parallelisation becomes less efficient, regardless of the pattern detection method employed. This is attributed to the increasing computational cost of copying the lattice state to each OpenMP thread. The drop in efficiency is not as pronounced for rDFS-mdn as it is for the other algorithms, but still rDFS-mdn yields much slower KMC execution for any given number of threads.

The acceleration factors yielded by using RI are modest but still significant.

A qualitative change in behaviour is observed upon increasing the lattice size further to 840×840. Parallelisation no longer significantly accelerates the simulation, and, in fact, begins to slow it down when the number of threads is increased from 6 to 12. This suggests that copying the lattice state to each OpenMP thread becomes a simulation bottleneck. This is corroborated by the fact that, with 12 threads, the KMC execution speed becomes roughly equal for all three subgraph isomorphism algorithms. It is thus clear that shared-memory parallel processing may not be worthwhile when the lattice is particularly large, and users of *Zacros* should carry out their own short benchmarks to determine the optimal simulation setup for a given system. To tackle large lattices, we have recently implemented distributed-memory parallelisation in *Zacros*, coupling the graph-theoretical KMC approach with the Time-Warp algorithm.[2]

References

[1] Srikanth Ravipati, Mayeul D'Avezac, Jens Nielsen, James Hetherington, and Michail Stamatakis, "A Caching Scheme to Accelerate Kinetic Monte Carlo Simulations of Catalytic Reactions", *J. Phys. Chem. A* **124**(35), pp. 7140 (2020).

[2] Srikanth Ravipati, Giannis D. Savva, Ilektra Athanasia Christidi, Roland Guichard, Jens Nielsen, Romain Réocreux, and Michail Stamatakis, "Coupling the time-warp algorithm with the graph-theoretical kinetic Monte Carlo framework for distributed simulations of heterogeneous catalysts", *Comput. Phys. Commun.* **270**, pp. 108148 (2022).
